# A Functional Proteomic Method for Biomarker Discovery

**DOI:** 10.1371/journal.pone.0022471

**Published:** 2011-07-19

**Authors:** Fred Reynolds, Nivedha Panneer, Christopher M. Tutino, Michael Wu, William R. Skrabal, Christopher Moskaluk, Kimberly A. Kelly

**Affiliations:** 1 Department of Biomedical Engineering, University of Virginia, Charlottesville, Virginia, United States of America; 2 Cardiovascular Research Center, University of Virginia, Charlottesville, Virginia, United States of America; 3 Department of Pathology, University of Virginia, Charlottesville, Virginia, United States of America; 4 Department of Biochemistry and Molecular Genetics, University of Virginia, Charlottesville, Virginia, United States of America; National Institutes of Health, United States of America

## Abstract

The sequencing of the human genome holds out the hope for personalized medicine, but it is clear that analysis of DNA or RNA content alone is not sufficient to understand most disease processes. Proteomic strategies that allow unbiased identification of proteins and their post-transcriptional and -translation modifications are an essential complement to genomic strategies. However, the enormity of the proteome and limitations in proteomic methods make it difficult to determine the targets that are particularly relevant to human disease. Methods are therefore needed that allow rational identification of targets based on function and relevance to disease. Screening methodologies such as phage display, SELEX, and small-molecule combinatorial chemistry have been widely used to discover specific ligands for cells or tissues of interest, such as tumors. Those ligands can be used in turn as affinity probes to identify their cognate molecular targets when they are not known in advance. Here we report an easy, robust and generally applicable approach in which phage particles bearing cell- or tissue-specific peptides serve directly as the affinity probes for their molecular targets. For proof of principle, the method successfully identified molecular binding partners, three of them novel, for 15 peptides specific for pancreatic cancer.

## Introduction

While the sequencing of the human genome was a great advance for many areas of biology, the many steps between DNA and protein, each of which is involved in complex, incompletely understood feedback loops, make the link between them often non-linear. For example, almost all sufferers of Down syndrome share the same genetic flaw (trisomy 21), but the degree of impairment varies greatly between individuals [1]. A study examining closely related species shows small differences in DNA sequence can become amplified in protein amino acid sequences, with even greater differences in protein expression levels [2]. These suggest that a true understanding of a disease state will be more accurate when both genomic and proteomic data is taken into account.

Unfortunately, studying the total protein expression of a cell is a difficult task. The human genome codes for approximately 38,000 proteins [3]. With multiple isoforms, multiple post translational modifications, and a 6 order of magnitude differences in expression levels, verifying the presence of the entire proteome, much less quantifying protein expression levels is beyond the ability of today's science. Restricting the proteomic study to the plasma membrane allows simplification of the method while retaining important information. Membrane proteins allow the cell to sense and manipulate its environment, and are indicative of processes in the cell. Resistance to chemotherapeutics by efflux requires transport proteins on the membrane [4], [5], metastasis is initiated by changes in the proteins that interact with the stroma [5]–[7], while cells undergoing growth and invasion express other markers [8]. The assumption is that any cellular behavior of interest will be reflected in the cell surface proteins, which greatly reduces the number of proteins to examine.

Standard proteomic methods of identifying relevant membrane proteins, such as biotinylation and capture [9], [10] can yield hundreds of proteins, most of which do not vary significantly in expression levels between the diseased tissue and healthy cells. Identifying and quantifying all these proteins on both the disease model and the negative cell line by mass spectroscopy is a very expensive and difficult undertaking typically performed in industry or highly specialized academic laboratories. Afterwards, the tedious task of comparing expression levels between the two data sets and discarding the vast majority of invariant and uninteresting proteins remains.

An alternative approach is to use screening methodologies such as phage display, aptamers, or carefully planned small molecule screens to probe the cell surface to identify functionally relevant proteins. Phage display in particular is the longest-standing platform amongst the display technologies with tens of thousands of publications. However, the screening method leads to compounds that bind to the proteins of interest, not the identity of the proteins themselves. Optimizing affinity chromatography, the standard method for determining the proteins phage derived peptides bind to [11]–[14], is time consuming, and must be done for each peptide clone. For a typical screen yielding several dozen peptide sequences, this method takes too long to be practical. Computer based methods, such as BLAST, yield a large number of non-statistically significant matches, with no guarantee that any are the real binding partner.

In this paper, we describe a functional proteomics method based on phage display screening and biochemical techniques. The described method is a robust, easy to use, and generally applicable approach in which phage particles bearing cell- or tissue-specific peptides serve directly as the affinity probes for their molecular targets. For proof of principle, we have used this method to identify the binding partners (three of which are novel biomarkers) of 15 peptides from a phage display screen that are specific for pancreatic cancer [15].

## Materials and Methods

### Cell culture

L3.6pl cells, a gift from professor Todd Bauer, were cultured in MEM with 10% fetal bovine serum, 1% l-glutamine, and 1% penicillin/streptomycin solution using established protocols.

### Phage Labeling

Phage were grown to a concentration of at least 10^10^ pfu/µL and suspended in 1 mL of PBS. Sulfo-SAED (Thermo Scientific, Rockford, IL), 2.5 mg and NHS-biotin (Thermo Scientific, Rockford, IL), 0.5 mg, were dissolved in 50 µL dimethyl sulfoxide and added to the phage. The reaction proceeded for one hour at 4°C then the phage polyethyleneglycol (PEG) precipitated 3 times according to standard methods [16] and resuspended in 2 mL DPBS with 10% FBS. A negative control using M13KE phage (NEB, Ipswich, MA), which are devoid of display peptides was utilized. To determine the extent of phage labeling, a standard curve composed of dilutions of sulfo-SAED in water, starting at 1 µM were made and the fluorescence measured at 350 nm excitation, 450 nm emission for each concentration. These results were compared to the fluorescence of the labeled phage at 10^9^ PFU/µL and 10^8^ PFU/µL.

### Pulldown

To determine the phage affinity binding partner, L3.6pl cells were plated in 10 cm petri dishes and allowed to grow for at least 48 hours to reach 70–90% confluency. Cells were washed twice with DPBS, then subsequently incubated with the labeled phage solutions (PDAC selected from NEB PhD 7 M13 library or control phage M13KE) in the dark for one hour at 4°C. The phage were removed and the cells again washed 4–6× with DPBS+0.1% tween, placed on ice, and the sulfo-SAED crosslinker activated by 10 min exposure to UV light using a 30 watt 365 nm lamp (Spectroline model XX-15A). The cells were then lysed with 1% Triton X-100 in PBS with mammalian protease inhibitor added per manufacturer's instructions (Sigma, St Louis, MO). The cell lysates were incubated for 20 minutes with 40 µL of Dynal M-280 streptavidin beads (Invitrogen). The beads were washed several times with 10× PBS containing 1% Triton X-100. The beads were then quickly rinsed with 0.1 M pH 2.2 glycine, then the protein bound to the phage eluted by cleaving the crosslinker with 50 µL of the pH 2.2 glycine buffer containing large amounts of dithiothreitol (DTT). For analysis, half of the solution was run on a SDS-PAGE gel and stained with either Coomassie blue or a mass spectroscopy compatible silver stain. Bands excised from the SDS-PAGE gel were sent for tryptic digest/mass spectroscopy analysis at either Tufts University core facility or the University of Virginia mass spectroscopy core facility for identification. The remaining lysate was utilized for validation experiments (western blot) once the identity of the protein was determined by mass spectroscopy.

### ELISA

#### Protein based ELISA

Recombinant purified proteins (R&D systems (vimentin) and Abcam (pyruvate kinase M2 and Annexin A2)) were dissolved in PBS and plated onto Nunc Maxisorp plates (5 µg protein/well) overnight at 4°C. Adsorbed BSA (5%) or Annexin A2 (for non-Annexin binding clones) were used as a negative control. Wells were washed, blocked with casein, then incubated with 50 µL phage (10^9^ pfu/µL) for 30 min at 37°C. After incubation, wells were again washed 6× times with 1% Tween in PBS, then incubated with anti-M13 antibody-HRP conjugate (GE healthcare) (1∶3000 dilution in PBS) for 1 hr at 37°C. Plates were again washed and developed with TMB then absorbance read on an absorbance plate reader (Molecular Devices).

#### Cell based ELISA

Six wells of a 96 well plate were L3.6pl cells (20,000 cells/well). Once cells reached 70–80% confluence, they were washed three times with DPBS+Mg^2+^ and Ca^2+^ for five minutes each. Primary antibodies (Abcam ab75933 (Annexin A2) and Novus Biologicals NBP1-39660 (Pyruvate Kinase M2)) were diluted to 500 nM in 100 uL DPBS+Mg^2+^ and Ca^2+^ with 1% BSA and incubated on ice with the cells in triplicate. For a negative control, cells were incubated on ice with 100 uL DPBS+Mg^2+^ and Ca^2+^ with 1% BSA. The wells were then washed three times with DPBS+Mg^2+^ and Ca^2+^ for five minutes each. Secondary antibodies anti-Rabbit IgG (GE Healthcare NA9340V (Annexin A2) and anti Goat IgG (R&D Systems HAF109 (Pyruvate Kinase M2)) were diluted 1∶1000 in 100 uL DPBS+Mg^2+^ and Ca^2+^ with 1% BSA and incubated with all wells for 30 minutes on ice. Subsequent to incubation, the wells were then washed three times with DPBS+Mg^2+^ and Ca^2+^ for five minutes each. 100 uL of TMB was added to each well and the plate incubated at RT for 15 minutes. Absorbance was then read on an absorbance plate reader at a wavelength of 650 nm.

### Cell fractionation

L3.6pl cells grown to 80–90% confluency in a 10 cm petri dish were washed several times with PBS, then mixed with 5 mL hypotonic lysis buffer (10 mM HEPES, pH 7.4, 15 mM KCl, 2 mM MgCl_2_ and protease inhibitors). The cells were scrapped off the dish then transferred to an Eppendorf tube where they were incubated for 2 hours on ice. To remove cellular debris and pellet nuclei, the cells were then centrifuged at 1300 g for 5 min. The nuclear fraction was washed twice with PBS then saved for western blot analysis. The supernatant was centrifuged at 100,000 g for 30 min to separate the cell membrane fraction, which was washed 2× with water, from the cytoplasm fraction. Both were saved for western blot analysis.

### Tissue microarrays

Tissue microarrays were prepared and scored by the University of Virginia Biorepository and Tissue Research Facility. Needle biopsies of human cancer and normal controls were sectioned onto glass slides and antibody stained using protocols established by staining tissue sections known to express the antigen of interest. The cancers selected were the 20 of the most common lethal human carcinomas: 12 samples each of colon and rectum adenocarcinoma, prostate adenocarcinoma, pancreatic adenocarcinoma, ovarian papillary carcinoma, endometrioid carcinoma, and transition cell (urothelial) carcinoma; 11 cases of head and neck squamous cell carcinoma; 6 cases each of small cell carcinoma of the lung, lung adenocarcinoma, breast ductal carcinoma and lobular carcinoma, hepatocellular carcinoma, bile duct cholangiocarcinoma, esophageal adeno- and squamous cell carcinoma, and stomach intestinal and signet ring adenocarcinomas; 5 samples each of lung squamous cell carcinoma and clear cell renal carcinoma; and three samples each of papillary and chromophobe type renal carcinoma. The stained tissues were graded by a pathologist for number of cells stained (0–3) and intensity of staining (0–3), and the two numbers multiplied together. The stained tissues were graded by a pathologist for number of cells stained (0, 1 = <25%, 2 = 25–75%, 3 = >75%) and intensity of staining (0, 1 = weak, 2 = moderate, 3 = strong), and the two numbers multiplied together for an index score.

The tissue used in the immunohistochemical stains were obtained retrospectively from archival clinical tissue samples by the UVA Biorepository and Tissue Research Facility (BTRF), with review and authorization by the UVA IRB (Protocol #13281). The tissue was used to create a tissue microarray (TMA) with samples identified by a research code created by the BTRF. The TMA was provided to the investigators thus de-identified and used in this study with separate UVA IRB authorization (Protocol # 13310).

## Results

**Figure 1:**
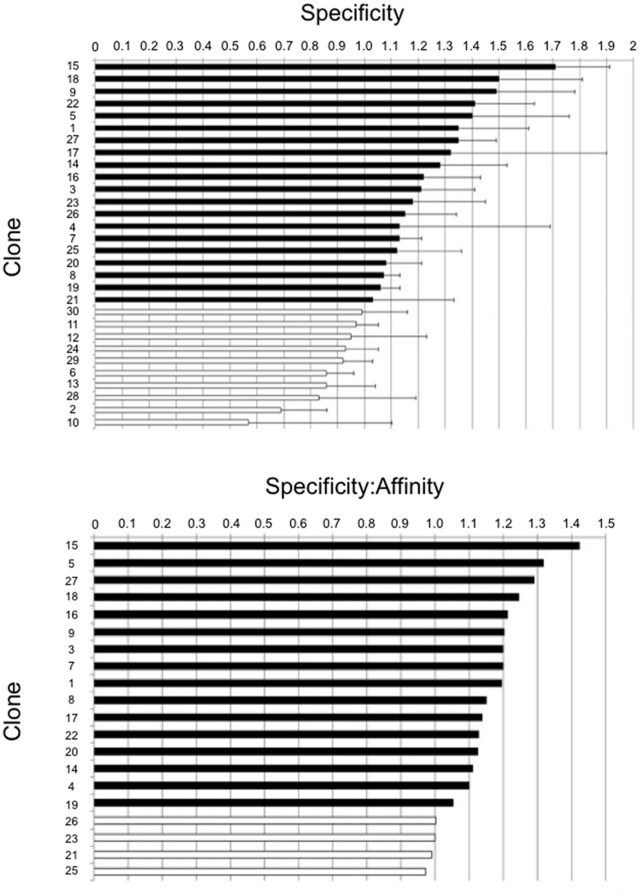
Ranking of clones for selection. A) Specificity of each clone as measured by ELISA. Clones in white were insufficiently specific (>1) and therefore, not used. B) Weighted sum of the specificity and affinity of each clone. Clones in white were not investigated further.

As proof of principle, we determined the surface markers of pancreatic cancer from a previously published selection for phage that specifically bind pancreatic cancer cells culled from transgenic animals and also human derived PDAC cell lines [15]. Not all of the 30 phage clones selected have ideal binding properties; phage clones with poor specificity are unlikely to yield relevant proteins and poor affinity will make it difficult to identify a protein binding partner. To reduce the 30 selected clones to the most promising, the phage clones were first ranked by specificity (defined as the ratio of binding to target cell line vs a background cell line)(Fig. 1A); those with ratios at or below one were removed from consideration, discarding 33% of the phage clones. To further refine the rankings, the weighted sum of the specificity and affinity was used (Fig. 1B). Specificity was weighted more heavily than affinity because the relevance of the binding partner of clones of poor specificity is suspect. Phage with a combined weighted average of less than 1 were discarded.

**Figure 2:**
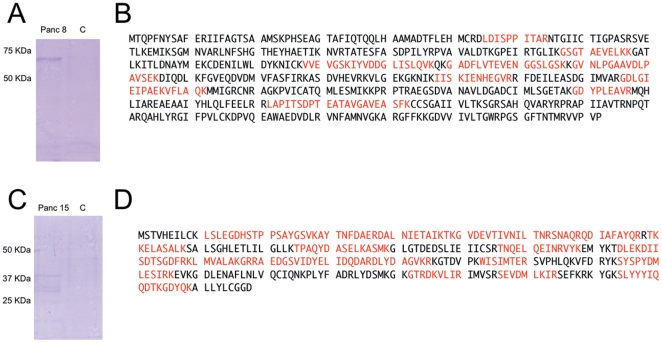
Pulldown and mass spectroscopic analysis. Elution sample from Panc 8 (A) and Panc 15 (C) were run on SDS/PAGE then stained with coomassie. Sequence of pyruvate kinase M2 (B) and Annexin A2 (D), with peptides recovered from tryptic digest of the band from (A) and (C) in red.

**Figure 3:**
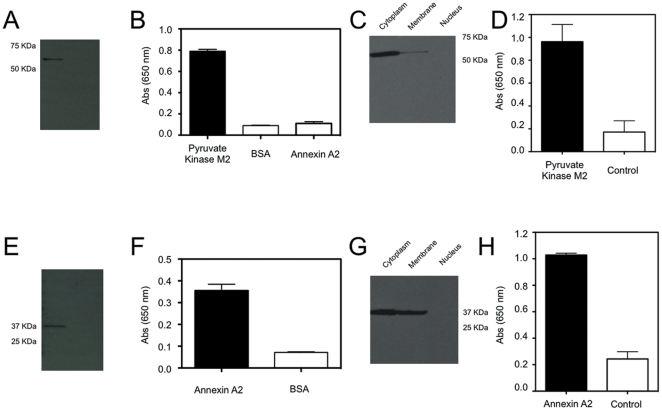
Validation of affinity partner for phage clones. A) Western blot of the protein that binds to clone 8, probed using anti-pyruvate kinase M2 antibody. B) ELISA of clone 8 incubated with purified pyruvate kinase M2, or with BSA or recombinant annexin A2 as negative controls. C) Western blot of cell fractionation using anti-pyruvate kinase M2 antibody D) ELISA on intact, non-permeabilized L3.6pl cells with anti-pyruvate kinase M2 antibody. E) Western blot of clone 15 associated protein probed with anti annexin A2 antibody. E) ELISA showing binding of clone 15 to annexin A2 protein. F) Western blot of cell fractionation using anti annexin A2 antibody. G) ELISA on intact, non-permeabilized L3.6pl cells with anti-Annexin A2 antibody.

The methodology for identifying the cell-surface binding partners of the phage is very similar to an immunoprecipitation. Phage were labeled with biotin and sulfo-SAED, a photoactive crosslinker. Loading of sulfo-SAED was characterized by fluorescence spectroscopy to be 660 photolinkers/plaque forming unit. These labeled phage clones were incubated with the cells then photolysed to activate the sulfo-SAED; crosslinking the phage with the protein they bind to. The cells were lysed, the phage and protein extracted with streptavidin beads and the cross linked protein released by disulfide cleavage, then analyzed by SDS/PAGE. Gel analysis demonstrated that Clone 8 has one major band at 60 KDa (Fig. 2A). Tryptic digest of the band followed by mass spectroscopic analysis identified pyruvate kinase M2 as the protein present in the analyzed band (Fig. 2B). In contrast Clone 15 yielded a band at 37 KDa (Fig. 2C) that upon analysis was revealed to be annexin A2 (Fig. 2D). To confirm the mass spectroscopy data, the samples from the pulldowns were examined by western blotting (Fig. 3A and 3E). As a further independent confirmation of the protein binding partner of the phage clones, ELISA was used to examine the phage binding to recombinant proteins (Fig. 3B and 3F). For example, phage clone 8 had a 9-fold higher binding to pyruvate kinase M2 when compared with BSA. In addition, clone 15 was specific to Annexin A2 as it had 5 fold higher binding when compared with BSA. A few phage clones were cross examined against proteins other than BSA that did not match their binding partner to control against general non-specific binding to other proteins (Fig. 3B).

Several of these proteins have not been reported on the cell membrane in pancreatic cancer, and their known functions are not compatible with location on the cell surface. Therefore, to test whether the identified proteins are present on the membrane, cells were fractionated into cytoplasm, membrane, and nuclear fragments and examined by western blot. In all the clones examined, the protein was present in the membrane fraction as demonstrated in Fig. 3C and 3G. An ELISA was performed on intact, non-permeabilized cells to further determine the presence and accessibility of the proteins on the cell membrane. L3.6pl cells were incubated with Annexin A2 antibody, pyruvate kinase M2 antibody or secondary only. Annexin A2 antibody had a 4.2 fold higher binding to cells when compared with secondary antibody alone (Fig. 3D). Similarly, pyruvate kinase M2 had a 5.6 fold higher binding to cells when compared with secondary antibody alone demonstrating (Fig. 3H) the accessibility of the proteins on the cell surface.

Of the 30 clones identified in the original screen, 16 were retained for further analysis after removing clones with inadequate affinity and specificity, for which we were able to identify 15 affinity binding partners (Table 1). Each of these clones except 9 and 14 were validated by ELISA against recombinant protein to verify the protein binding partner. The exceptions bind to a specific isoform of histone H1 that we were unable to obtain as a recombinant protein. The gels from clones that bound to the same protein all had very similar appearances; for example, all the annexin A2 binding clones gave gels that looked like that of panc 15 (fig. 2C). In almost all cases, the strongest band was the protein the phage bound to as validated by western blot and phage ELISA against purified protein.

**Table 1:** Listing of the phage clones, their binding peptide sequence, and their associated targeted protein.

Clone	Sequence	Affinity ligand
Panc 3	VSQTLRL	Annexin A2
Panc 4	GSLYPTA	Annexin A2
Panc 15	TMAPSIK	Annexin A2
Panc 19	QTLPKLY	Annexin A2
Panc 20	RLAPIN	Annexin A2
Panc 22	VNDRNVK	Annexin A2
Panc 5	QSPDEVW	Vimentin
Panc 7	WMHQPTY	Vimentin
Panc 16	AKSSLNS	Vimentin
Panc 17	TQHQVTA	Vimentin
Panc 18	APWTHNS	Vimentin
Panc 8	TGTAYPY	Pyruvate kinase M2
Panc 9	LKPTHHA	Histone H1
Panc 14	YATHHNT	Histone H1
Panc 27	KTLLPTP	Plectin 1

**Figure 4:**
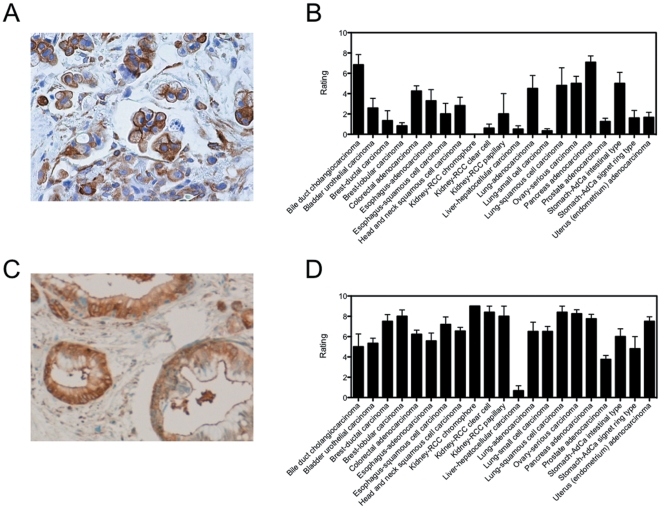
Tissue microarray data. Values are pathologist's scoring of number of cells stained (0–3) and intensity of staining (0–3) multiplied together. A) Representative tumor section stained for plectin. Note the membrane staining. B) Pathologist's scoring of human cancer biopsy specimens stained for plectin. C) Representative PDAC tumor biopsy section stained for pyruvate kinase M2. D) Pathologist's scoring of pyruvate kinase M2 stained human cancer biopsy tissue sections.

While these proteins were verified to be associated with the surface of the L3.6pl cell line, we sought to determine the potential relevance to pancreatic cancer of selected proteins through immunohistochemistry. Antibodies to either pyruvate kinase M2 or plectin were used to determine the expression levels and extent of staining in a series of human biopsy specimens (Fig. 4). These were chosen as neither have been previously reported in pancreatic cancer whereas Annexin A2 is a known protein expressed in pancreatic cancer. As the kRAS mutation that initiates pancreatic cancer [17] is common to many other cancers [18], the immunohistochemistry was conducted in a tissue microarray format with 20 of the most common lethal human cancers (n = 3–12 patients for each cancer). A pathologist ranked the stained tissues for percentage of cells stained (0–3) and intensity of staining (0–3), with the average product of these shown in Fig. 4. Plectin shows strong membrane staining in pancreatic cancer (Fig. 4A) and is significant in other cancers including bile duct cholangiocarcinoma, lung adenocarcinoma, lung squamous cell carcinoma, ovarian cancer, and intestinal type stomach cancer (Fig. 4B). Closer examination of a representative pancreatic cancer sample (Fig. 4A) shows cytoplasmic and membrane staining, consistent with the cell fractionation data [15]. Pyruvate kinase M2 also showed strong staining in pancreatic cancer and almost all other cancer types (Fig. 4D), with both cytoplasmic and membrane staining (Fig. 4C). These data suggest that both of these may be potential markers of pancreatic cancer and other cancers as well.

## Discussion

Proteomic based strategies that provide for the unbiased identification of molecules and importantly their post-transcriptional and post-translational modifications provide valuable information and are an important complement to genomic strategies. However, the enormity of the proteome and limitations in proteomic methods make it difficult to determine the targets that are particularly relevant to human disease. Therefore, we have developed a functional proteomics method based on phage display screening and biochemical techniques to fill this void. The described method is robust, easy to use, and a generally applicable approach to other diseases, tissues, and potentially other screening platforms. In this method, the selected phage clones, which are cheap and easy to produce, are used directly as the probes, bypassing many of the steps required for other partner identification strategies. Further, by including the crosslinking step, harsh detergents and conditions sometimes necessary to extract transmembrane proteins from the membrane but would break the protein interaction can be used and thus, these binding partners can be identified

It is remarkable that the phage particles do not crosslink randomly to surface proteins (Fig. 2A). The particle is 1 um long, with hundreds of photoactivatable crosslinkers arrayed randomly along its entire length. The specific target-binding peptide is at one tip of that particle, 1 um away from the most distant crosslinkers. If a target-bound particle were able to lie parallel to the cell surface, it could potentially be crosslinked to any surface protein within a 1-um radius of that specific target. Presumably, the population of proteins within such a large radius is indistinguishable from a random sampling of all the cell's surface molecules. According to this scenario, therefore, all clones should crosslink to a random sampling of surface molecules, irrespective of the binding specificity of their displayed peptides. Our results are entirely inconsistent with this expectation: different phage clones crosslink to just one or a few surface proteins, and those proteins differ from clone to clone. Moreover, the binding specificity of the displayed peptides has been corroborated in multiple ways that are completely independent of crosslinking. In light of these considerations, we favor an alternative scenario, according to which target-bound particles are constrained to lie perpendicular to the cell surface, so that only crosslinkers within a few nanometers of the target-binding peptide are close enough to crosslink to surface proteins. Within such a narrow radius of reactivity, it is entirely plausible that the actual target protein predominates, in full accord with our results. In support of this scenario is the fact that the surfaces of both the phage particle and the cell are negatively charged, and thus might well repel each other except where specific target binding overcomes the repulsion.

For proof of principle, we have utilized the phage clones identified from a previously performed phage display screen for peptides specific to pancreatic cancer. Of the 16 phage clones that made biological sense ie. had specificity and high affinity for pancreatic cancer, we were able to identify the binding partner of 15 of the phage clones. The proteins identified consist of known and interestingly, novel surface markers (Table 1). Vimentin [19]–[21] and annexin A2 [22]–[28], the two proteins with the largest number of associated phage clones, have been shown in the literature to be on the cell surface of pancreatic cancer. Vimentin is an intermediate filament protein; part of the cytoskeleton of the cell [21], and is a marker of the epithelial-mesenchymal transition [19], [20]. Annexin A2 is found in all compartments of the cell [23], [26], [29] and has a vast array of functions and binding partners. It is involved in DNA replication [29], and invasion and metastasis [23], [26]–[28], [30], [31]. It binds with gastrin [32], tissue plasminogen activator [27], [28], actin [33], and tenacin C [23] and has been investigated as a serum [34] and urine [35] biomarker of pancreatic cancer. Expression levels of annexin A2 are related to resistance to gemcitabine [36], which is mediated by the interaction of an alternatively spliced segment of tenascin-C with annexin A2 under the control of the PI3K/Akt and NF-kB pathways [37]. While these effects are shown from whole cell lysates of annexin A2, if the same relationship of high expression correlating with gemcitabine resistance holds on the cell surface, we have identified 6 peptides that can be used to probe this phenomenon.

Pyruvate kinase M2 isoform is an embryonicly expressed enzyme not normally found in healthy individuals. However, it is the sole isoform expressed in most forms of cancer [38], even though it is less efficient than other isoforms. It has been investigated as a serum tumor marker [39]–[41], but only two contradictory reports mention surface expression [42], [43] in pancreatic cancer with one demonstrating surface expression [43] and the other absence of surface expression [42]. However, the paper reporting absent surface expression also claims to find the protein expressed in the normal pancreas and in chronic pancreatitis [42]. Our work demonstrated strong expression in 19 of 20 cancers examined including pancreatic cancer with only hepatocellular carcinoma devoid of any staining.

Interestingly, we identified histone H1 as being present on the cell surface, an unexpected finding given the function of histones and the observation that histones present in cell culture media will cross the cell membrane and travel to the nucleus [44]–[47]. The function of histone HI is to sequester the ends of DNA into the nucleosome and link nucleosomes together. Further literature searching however, shows that histone H1 was reported on the surface of two melanoma cell lines [9] and on neurons [48] and Schwann cells [48]. Apoptotic cells [49]–[51] and many cells of the immune system [52]–[57] express nucleosomes, including histone H1, on the cell surface, which bind to various compounds such as sulfonated polysacharaides [56], [57], plasminogen [53], [58] and thyroglobulin [52], and act as antimicrobial agents [59]. To our knowledge, this is the first report of the presence of histone H1 on the surface of pancreatic cancer. Its role and function in pancreatic tumorigenesis and or metastasis is currently unknown. However, due to the immune system expression, histone H1 maymake a poor target for either imaging or drug delivery.

Another interesting novel pancreatic cancer specific protein, plectin, was identified through our method. In normal epithelial cells, predominately skin and muscle cells, plectin is a major component of the hemidesmosome- linking the cell to its basement membrane. Patients that have mutations in plectin have a severe skin blistering disease, epidermolysis bullosa, underscoring the importance of plectin to the hemidesmosome and cell-cell junctions. When plectin is expressed in cells, it is found in the cytoplasm. However, in pancreatic cancer, it is expressed both in the cytoplasm and on the cell surface. Recently, plectin was shown to be expressed in all pancreatic cancers examined [15], [60], but has no expression in the healthy pancreas or in other benign conditions. Experiments are underway to determine the mechanism of the cell surface population of plectin and its potential role in pancreatic cancer.

Of the 16 phage clones examined, it proved possible to determine the binding partner of 15, a success rate of ∼94%. We were unable to find the affinity partner of the 16^th^ clone, clone 1, despite its good specificity and affinity, even after several attempts. There are two possible reasons for this. The original screen was conducted on cells derived from a mouse model of pancreatic cancer [15], as this allowed access to needed normal pancreatic ductal cells that otherwise would be very difficult to obtain. The affinity binding partners were determined on a human cancer cell line, as we are interested in biomarkers for human disease. It is possible that clone 1 binds solely to a mouse protein and does not cross-react with the human variant. Alternatively, the protein that clone 1 binds to is an artifact of the cell line the screen was conducted on and is not general to pancreatic cancer in humans.

Here we report a new method of cell surface proteomic analysis using phage to probe the cell surface followed by identification of the protein the various phage clones bind to. The method gives a snapshot of the most abundant proteins on the membrane that are overexpressed due to disease and have identified several proteins, which upon further study, may give important insights into pancreatic cancer. Further, the original phage display screen identified peptides that can then be converted into imaging agents that may aid in diagnosis and targeted drug delivery for pancreatic cancer.
